# Distribution and Maintenance of Histone H3 Lysine 36 Trimethylation in Transcribed Locus

**DOI:** 10.1371/journal.pone.0120200

**Published:** 2015-03-16

**Authors:** Henel Sein, Signe Värv, Arnold Kristjuhan

**Affiliations:** Department of Cell Biology, Institute of Molecular and Cell Biology, University of Tartu, Riia 23, Tartu, 51010, Estonia; CNRS, FRANCE

## Abstract

Post-translational modifications of core histones play an important role in the epigenetic regulation of chromatin dynamics and gene expression. In *Saccharomyces cerevisiae* methylation marks at K4, K36, and K79 of histone H3 are associated with gene transcription. Although Set2-mediated H3K36 methylation is enriched throughout the coding region of active genes and prevents aberrant transcriptional initiation within coding sequences, it is not known if transcription of one locus impacts the methylation pattern of neighbouring areas and for how long H3K36 methylation is maintained after transcription termination. Our results demonstrate that H3K36 methylation is restricted to the transcribed sequence only and the modification does not spread to adjacent loci downstream from transcription termination site. We also show that H3K36 trimethylation mark persists in the locus for at least 60 minutes after transcription inhibition, suggesting a short epigenetic memory for recently occurred transcriptional activity. Our results indicate that both replication-dependent exchange of nucleosomes and the activity of histone demethylases Rph1, Jhd1 and Gis1 contribute to the turnover of H3K36 methylation upon shut-down of transcription.

## Introduction

In eukaryotic cells, genomic DNA is packaged into a nucleoprotein structure known as chromatin. Nucleosome, a fundamental repeating unit of chromatin, consists of 146 base pairs of DNA wrapped around an octameric core composed of two molecules of each histone protein: H3, H4, H2A and H2B [1]. Compact chromatin structure forms a natural barrier to many DNA-related processes, including transcription. Numerous mechanisms influence the structure of chromatin in order to gain access to DNA, such as histone modifications [2], chromatin remodelling [3] and histone eviction [4]. Covalent modifications are crucial to the regulation of chromatin dynamics by altering the properties of chromatin and regulating the recruitment of additional effector proteins to chromatin [5]. One such post-translational modification is histone methylation that occurs on both lysine and arginine residues of histones H3 and H4. In budding yeast, *Saccharomyces cerevisiae*, histone H3 lysines 4, 36 and 79 (H3K4, H3K36, H3K79) are methylated by histone methyltransferases Set1, Set2 and Dot1, respectively [6–8]. Set2-mediated H3K36 methylation is one of the major histone modifications conserved in eukaryotes. Genome-wide chromatin immunoprecipitation assays have shown that H3K36 methylation is present in the open reading frames (ORF) of protein-encoding genes and trimethylation of H3K36 (H3K36me3) displays a positive correlation with transcription rates [9]. Set2 is recruited to genes through interactions with the phosphorylated repeats of the RNA polymerase II C-terminal domain (RNAPII CTD) [10, 11]. H3K36 methylation in turn is recognized by Eaf3 and Rco1 subunits of Rpd3S deacetylase complex, which restores the compact chromatin structure in the wake of elongating RNAPII and therefore represses cryptic transcription [12]. H3K36me3 also suppresses histone exchange and interaction of H3 with histone chaperons over coding regions [13]. Furthermore, for proper H3K36 di- and trimethylation, specific motifs within Set2 interact with histones H4, H2A and H3 [14–16].

It was long thought that histone methylation is a static modification, however the identification of the first histone demethylase LSD1 (lysine-specific demethylase 1), revealed methylation to be reversible and dynamic epigenetic mark [17, 18]. Recently, a family of histone demethylases characterized by the presence of a highly conserved Jumonji (JmjC) domain was reported [19, 20]. Among the five JmjC domain-containing demethylases identified in *Saccharomyces cerevisiae*—Rph1, Jhd1 and Gis1 have been associated with H3K36 demethylation [21–24].

Although genome-wide mappings of histone modifications have confirmed a strong correlation between active transcription and H3K36me3, the dynamics and turnover mechanisms of this modification are not fully understood. For example, it is not clear whether an active transcription in one locus could influence the modification of H3K36 in neighbouring chromatin domains and what are the mechanisms restricting the spreading of H3K36me3. Also the dynamics and mechanisms of H3K36 demethylation after transcription repression are not entirely clear. To clarify the mechanisms regulating H3K36 methylation and demethylation in chromatin, we investigated the dynamics of H3K36 trimethylation in an inducible-repressible gene locus. This experimental system allows rapid shut-down of gene transcription and the turnover of H3K36me3 can be monitored in the absence of continuous transcriptional activity in the locus. We show that H3K36 methylation is brought to the gene in transcription-coupled manner and the modification does not spread outside of the actively transcribed locus. Also, we demonstrate that turnover of the H3K36 methylation mark is relatively slow taking more than 60 minutes after transcription termination. Our data suggests that both replication-dependent exchange of histones and passive demethylation of K36 by histone demethylases contribute to the erasure of H3K36 methylation mark upon shut-down of transcription.

## Materials and Methods

### Yeast Strains

All *Saccharomyces cerevisiae* strains were congenic with strain W303 and are given in Table 1. The *GAL-VPS13-*3kb-term (AKY212) strain contains the terminator sequence of *FBA1* gene at 3220 bp downstream from the *VPS13* start-codon [25]. AKY212 expresses H3 with C-terminal E2 tag (GVSSTSSDFRDR) and is used as a wild-type yeast strain in this study. Demethylase deletion mutant strains (AKY752, AKY753, AKY754) were derived from AKY212 by transplacement of the open reading frames of *RPH1*, *JHD1* and *GIS1* genes with hphMX, natMX6 and kanMX markers, respectively. These strains in turn were used to create the deletion mutant strain of all three demethylases (AKY975). The overall fitness of deletion strains was similar to wt, since the deletion mutants grew at the same rate as wt strain at different temperatures and carbon sources (S1 Fig.). In strains AKY968 and AKY1142 the *BAR1* gene was deleted to achieve efficient α-factor arrest. AKY979 strain contains triple E2 tag in the C-terminus of Set2.

**Table 1:** Yeast strains.

Strain	Genotype	Source
AKY212	*W303 MATa GAL-VPS13*::*TRP1 hht1-hhf1Δ*::*LEU2 HHT2-E2tag*::*URA3 FBA1 term region at 3 kb in VPS13*	[25]
AKY752	*W303 MATa GAL-VPS13*::*TRP1 hht1-hhf1Δ*::*LEU2 HHT2-E2tag*::*URA3 FBA1 term region at 3 kb in VPS13 rph1Δ*::*hphMX6*	This study
AKY753	*W303 MATa GAL-VPS13*::*TRP1 hht1-hhf1Δ*::*LEU2 HHT2-E2tag*::*URA3 FBA1 term region at 3 kb in VPS13 jhd1Δ*::*natMX6*	This study
AKY754	*W303 MATa GAL-VPS13*::*TRP1 hht1-hhf1Δ*::*LEU2 HHT2-E2tag*::*URA3 FBA1 term region at 3 kb in VPS13 gis1Δ*::*kanMX*	This study
AKY968	*W303 MATa GAL-VPS13*::*TRP1 hht1-hhf1Δ*::*LEU2 HHT2-E2tag*::*URA3 FBA1 term region at 3 kb in VPS13 bar1Δ*::*hphMX6*	This study
AKY975	*W303 MATa GAL-VPS13*::*TRP1 hht1-hhf1Δ*::*LEU2 HHT2-E2tag*::*URA3 FBA1 term region at 3 kb in VPS13 rph1Δ*::*hphMX6 jhd1Δ*::*natMX6 gis1Δ*::*kanMX*	This study
AKY979	*W303 MATa GAL-VPS13*::*TRP1 MCM4-3xmyc tag*::*LEU2 SET2-3xE2tag*::*URA3 RPB3-3xE4tag*::*HIS3 FBA1 term region at 3 kb in VPS13*	This study
AKY1142	*W303 MATa GAL-VPS13*::*TRP1 hht1-hhf1Δ*::*LEU2 HHT2-E2tag*::*URA3 FBA1 term region at 3 kb in VPS13 rph1Δ*::*hphMX6 jhd1Δ*::*natMX6 gis1Δ*::*kanMX bar1Δ*::*HIS3*	This study

### ChIP Assay and Cell Cycle Arrest

Cells were grown overnight in yeast extract-peptone (YP) medium containing 2% glucose or 2% galactose as a carbon source. 15 ml of mid-log phase cells (200 ml for Set2 detection experiments) were fixed in 1% formaldehyde for overnight galactose and glucose timepoint samples for ChIP assays. Transcription repression was carried out by shifting overnight galactose-grown cells into medium containing 2% glucose. Samples were taken at different time points (5, 10, 15, 30, 60, 120 min) after shift to glucose and fixed for ChIP assay. For cell cycle arrest experiments, α-factor mating pheromone (GenScript) was used at the final concentration of 100 nM, and cell cycle status during the experiment was confirmed by flow cytometry analysis (S2 Fig.). ChIP assays were performed as described previously [26]. Whole cell extract from 10^7^ cells was used for ChIP assays with antibodies directed against anti-E2tag (5E11, Icosagen) or anti-histone H3 trimethyl-K36 (Abcam). Co-precipitated DNA was analysed by quantitative real-time PCR using ABI Prism 7900HT Fast Real-Time PCR System and LightCycler 480 Real-Time PCR System under standard conditions (40 cycles, 95°C 15s + 60°C 1 min). Maxima SYBR Green/ROX qPCR master mix (Thermo Scientific) was used. PCRs were done with primer pairs covering the coding region of *VPS13* and chromosome VI right arm telomere region for normalization. The *VPS13* primers were specific for the sequences at 2.6, 3, 3.6 and 4 kb downstream from the start-codon of *GAL-VPS13*. The sequences of primers are listed in the S1 Table.

## Results

**Fig 1:**
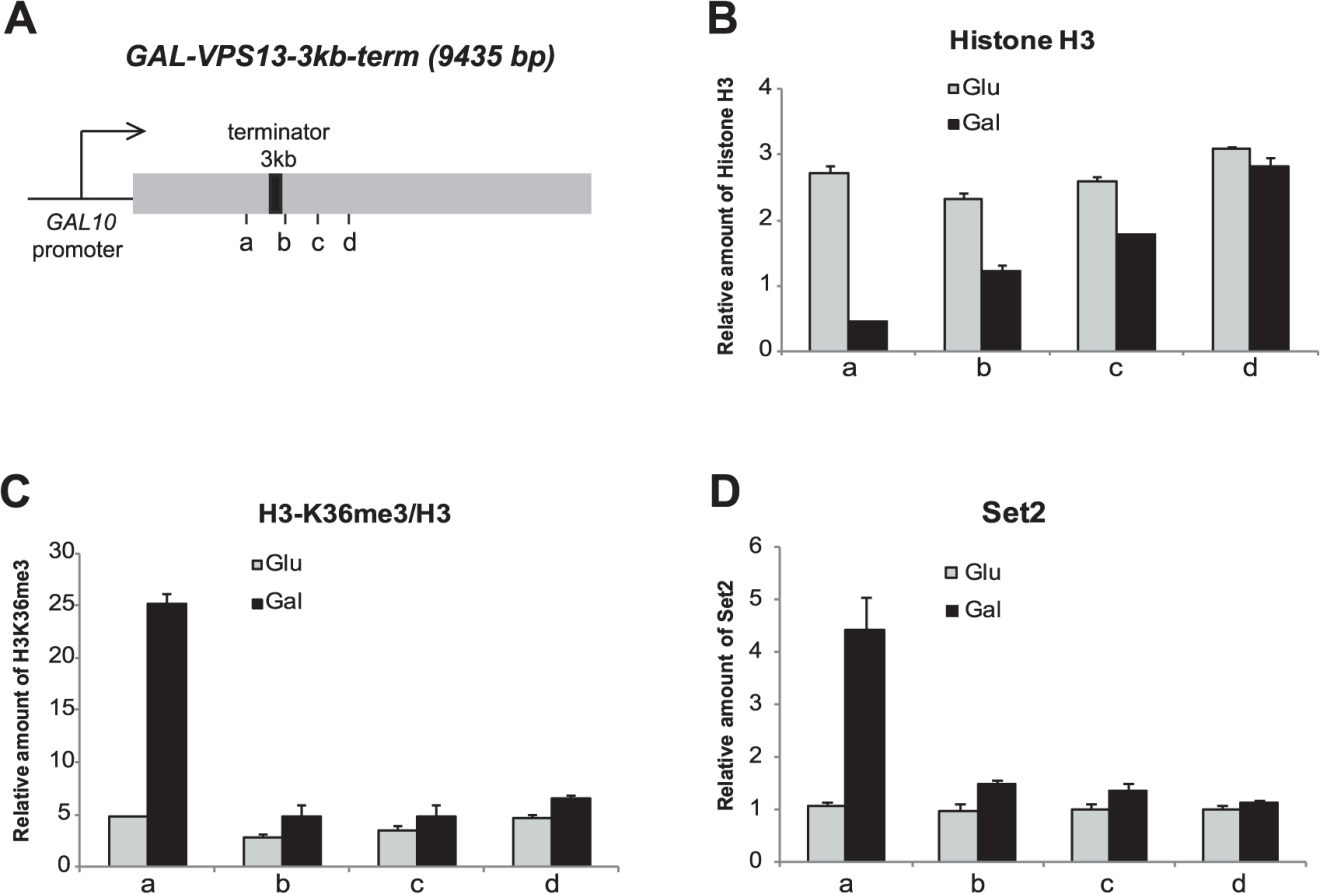
Distribution of H3K36me3 in *GAL-VPS13* locus. (**A**) Schematic representation of the 9435 bp long *GAL-VPS13* locus. The *GAL-VPS13-3kb–term* strain contains the *FBA1* transcription termination region inserted at 3 kb from the *VPS13* promoter (3 kb-terminator, black rectangle). Vertical lines beneath the gene indicate the positions of PCR probes 2.6 kb (a), 3 kb (b), 3.6 kb (c) and 4 kb (d) downstream from the start-codon of *GAL-VPS13*. (**B**) ChIP assay followed by qPCR was used to determine the relative amount of histone H3 in the coding region of *GAL-VPS13* upon transcriptional activation in galactose (black bars, Gal) and repression in glucose (grey bars, Glu). (**C**) The relative amount of H3K36me3 was determined in the coding region of *GAL-VPS13* upon transcriptional activation in galactose (black bars, Gal) and repression in glucose (grey bars, Glu). (**D**) The occupancy of Set2 upon transcriptional activation in galactose (black bars, Gal) and inactivation in glucose (grey bars, Glu) in the coding region of *GAL-VPS13*. In all assays, the ChIP signal obtained from nontranscribed region of the right arm telomere of chromosome VI (Tel6) was set as 1 and all samples are presented as relative to that. In addition, the H3K36 trimethylation signal in C was normalised to total H3 occupancy. Error bars show standard error of at least 3 independent experiments.

The genome of *S*. *cerevisiae* is relatively compact containing short genes and very little space between the ORFs. These features complicate the studies of transcription-dependent dynamics of histone modifications in yeast, as the chromatin modification pattern in any particular locus is likely to be influenced by the activity of several neighbouring genes. To overcome these difficulties, we used a 9.4 kb long *GAL-VPS13* gene with transcriptional terminator sequence inserted at 3 kb downstream of the start site (Fig. 1A). *GAL-VPS13* is under the control of *GAL10* promoter that makes it easy to induce and repress transcription with galactose or glucose in the growth medium, and the 6 kb long nontranscribed region of *VPS13* provides enough space to investigate the dynamics of chromatin modifications in the locus without significant influence from the neighbouring genes. We have previously shown that induction of *GAL-VPS13* leads to RNAPII-dependent disruption of nucleosomes in the locus and the normal nucleosomal structure is restored quickly after shut-down of transcription [25, 27]. We have also confirmed that the inserted transcriptional terminator ensures efficient termination of RNAPII in this region and leaves the *VPS13* coding region downstream of the terminator nontranscribed [25].

To determine the occupancy of nucleosomes and the distribution of H3K36 trimethylation in *GAL-VPS13* locus upon transcriptional activation, cells were grown overnight in the medium containing either glucose or galactose. In the glucose-containing medium the transcription of *GAL-VPS13* gene is repressed and the locus is packaged with nucleosomes as determined by the detection of E2-tagged histone H3 occupancy. In galactose-grown cells the *GAL-VPS13* locus is fully transcribed and disruption of the nucleosomal structure was observed upstream of the inserted transcriptional terminator site (Fig. 1B). H3K36 trimethylation was detected only before the terminator region, indicating that the distribution of H3K36 trimethylation was strictly restricted to the RNAPII-transcribed locus and did not spread to the areas beyond the terminator (Fig. 1C). Consistent with the H3K36 methylation pattern, we detected the H3K36-specific methyltransferase Set2 mainly in the transcribed area of *GAL-VPS13* (Fig. 1D).

**Fig 2:**
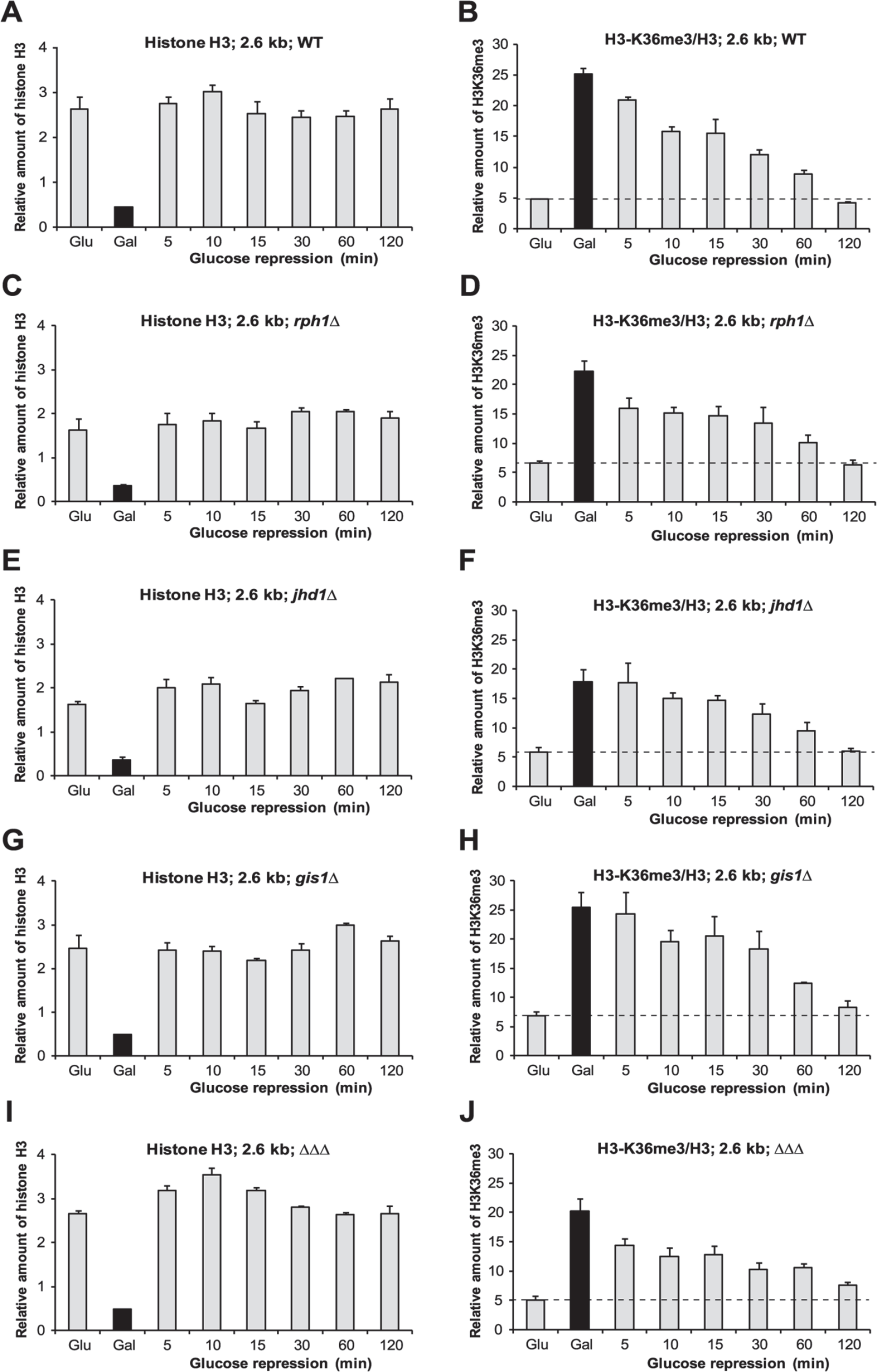
Dynamics of H3K36me3 in demethylase deletion strains. The relative amount of histone H3 (**A, C, E, G, I**) and H3K36me3 (**B, D, F, H, J**) was determined at 2.6 kb of the *GAL-VPS13* upon transcription repression in wild type (**A** and **B**), *rph1Δ* (**C** and **D**), *jhd1Δ* (**E** and **F**), *gis1Δ* (**G** and **H**) and *rph1Δjhd1Δgis1Δ* triple deletion mutant (ΔΔΔ **I** and **J**) strains. All samples were quantified as described in Fig. 1.

To study the maintenance of H3K36 methylation, cells were grown in YP-galactose medium for at least 15 hours and then shifted to YP-glucose medium to repress active transcription of *GAL-VPS13*. The persistence of H3K36 methylation in the locus was followed during the time-course of two hours at the coding region of *GAL-VPS13* before the terminator (2.6 kb). As expected, the nucleosomal structure in *GAL-VPS13* locus was recovered within minutes after transcriptional termination (Fig. 2A). However, the H3K36 trimethylation mark persisted in the locus at least 60 minutes after shut-down of transcription (Fig. 2B), indicating that after transcriptional inhibition, methylated histones stay in the composition of chromatin and mark the recently transcribed locus. The H3K36 trimethylation was reduced to the basal level after two hours of transcriptional repression (Fig. 2B). The relatively slow kinetics of H3K36me3 turnover suggests that demethylation of H3K36 is not actively regulated and occurs either by replacement of histones, or by non-targeted action of histone demethylases.

Demethylases Rph1, Jhd1 and Gis1 have been associated with histone H3K36 demethylation in actively transcribed genes. Although, their enzymatic activities are becoming clearer, the role of demethylases in regulation of transcription and chromatin structure is not fully understood. Rph1 has been shown to specifically target di- and trimethylation, while Jhd1 has been shown to demethylate mono- or dimethylated H3K36 [21–24, 28]. Structurally, Gis1 is similar to Rph1, but due to the differences in cofactor binding residues, it is predicted to have a modest to low demethylase activity [22]. To test whether histone demethylases affect the persistence of H3K36me3 in recently transcribed locus, we followed the turnover of H3K36me3 in strains where either *RPH1*, *JHD1*, *GIS1*, or all three of these were deleted. We could detect only minor differences in the dynamics of H3K36 demethylation in the *GAL-VPS13* locus when individual demethylases were deleted. In all strains, histones were reloaded to the locus immediately after gene repression and H3K36me3 persisted in the locus up to 60 minutes after transcription repression (Fig. 2C-H). Like in wt cells, the H3K36me3 was reduced nearly to the basal level at 120 minutes time-point in the strains with deletion of single demethylases, suggesting the redundancy of Rph1, Jhd1 and Gis1 in demethylation of H3K36. However, when all the three demethylases were deleted, the H3K36me3 mark was clearly detectable in *GAL-VPS13* locus 120 minutes after shut-down of transcription indicating the prolonged turnover of the modification (Fig. 2I and 2J). This suggests that all three demethylases are involved in proper turnover of H3K36me3, although their functions might be redundant. Possibly, the sequential or cooperative action of demethylases is required for the efficient demethylation of H3K36.

**Fig 3:**
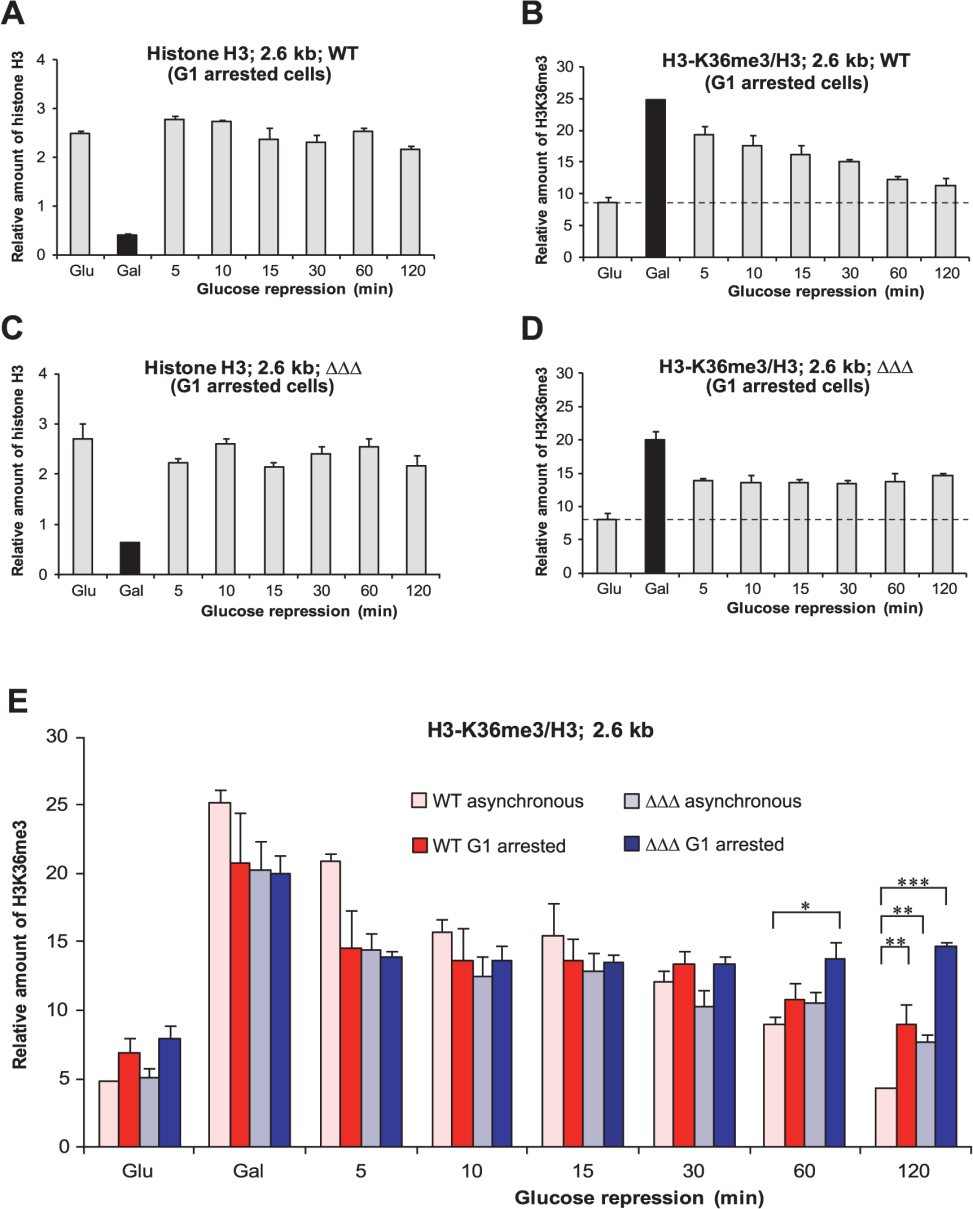
Replication-dependent loss of H3K36me3 after transcriptional repression in wt and *rph1Δjhd1Δgis1Δ* strains. The relative amount of histone H3 (**A** and **C**) and H3K36me3 (**B** and **D**) was determined upon glucose-mediated transcriptional repression at 2.6 kb in the coding region of *GAL-VPS13* in G1-arrested wt (**A** and **B**), and in *rph1Δjhd1Δgis1Δ* cells (ΔΔΔ; **C** and **D**). (**E**) Comparison of H3K36me3 turnover in wt and *rph1Δjhd1Δgis1Δ* strains grown asynchronously, or kept G1-arrested throughout the experiment. The Student *t* test was used to evaluate the statistical significance of differences between indicated samples. * indicates p<0.05; ** indicates p<0.005; *** indicates p<0.0001. All samples were quantified as described in Fig. 1. Cell cycle profiles of G1-arrested wt and *rph1Δjhd1Δgis1Δ* strains are shown in S2 Fig.

To investigate the role of replication-coupled exchange of histones in the removal of H3 methylation, we arrested cells in G1 and followed H3K36me3 turnover in the conditions where cells were unable to enter the S phase of the cell cycle. Cells were arrested in the late G1 phase with α-factor before and during repression of *GAL-VPS13* transcription, and the kinetics of H3K36me3 turnover was monitored. Similarly to unarrested cells the nucleosomal structure of the *GAL-VPS13* locus was quickly restored upon transcriptional inactivation (Figs. 2A and 3A). However, in G1-arrested cells the H3K36me3 mark was stable for longer period than in unarrested cells (Figs. 2B and 3B), indicating that in exponentially growing cells the replication-dependent exchange of histones contributes significantly to the erasure of H3K36me3 mark from recently transcribed locus. Our results suggest that both demethylation of H3K36 and replication-coupled exchange of nucleosomes contribute to the turnover of H3K36me3 upon repression of transcription. To test this hypothesis, we followed the dynamics of H3K36me3 turnover in G1-arrested *rph1Δ jhd1Δ gis1Δ* cells, where both proposed pathways for removal of H3K36me3 were eliminated. As expected, in G1-arrested *rph1Δjhd1Δgis1Δ* cells the nucleosomal structure of the *GAL-VPS13* locus was normally restored upon transcriptional repression (Fig. 3C) and the H3K36me3 modification stayed in the locus for more than two hours (Fig. 3D). The dynamics of H3K36me3 turnover in wt and *rph1Δ jhd1Δ gis1Δ* strains is summarized in Fig. 3E for better comparison of the results from asynchronously growing and G1-arrested cells.

## Discussion

H3K36me3 co-localizes with active transcription and H3K36 specific methyltransferase Set2 is brought to transcribed loci via its interaction with RNAPII [10, 11]. Once recruited to chromatin, Set2 binds to nucleosomes interacting with multiple residues of H2A, H3 and H4, and catalyses methylation of H3K36 [14, 15]. However, it is not known whether H3K36 methylation *in vivo* is limited to the histones located immediately in the transcribed locus, or can it spread over the nucleosomes, influencing the chromatin modification pattern in neighbouring loci. In the present study, we investigated distribution of histone H3K36 trimethylation in a transcribed locus and its turnover after transcriptional repression. We used a galactose-inducible *GAL-VPS13* locus to monitor the distribution of H3K36me3 in the region around the transcriptional terminator and found that H3K36 trimethylation was restricted to actively transcribed sequence and did not spread beyond transcribed area (Fig. 1). It has been shown that in nucleosome the residues H4 K44, H2A L116 and H2A L117 are critical for interaction with Set2 [14]. As all these residues lie in the core of the nucleosome, it is possible that transcription-coupled opening of chromatin and partial disruption of nucleosomes are required for efficient binding of Set2 to the locus. Therefore, nucleosomes in nontranscribed regions might be reluctant for Set2 recruitment, which in turn restricts the spreading of H3K36me3 into transcriptionally inactive loci.

Upon repression of transcription, H3K36 trimethylation was retained in the locus at least for 60 minutes (Fig. 2). The relatively slow kinetics of H3K36me3 turnover suggests that histone demethylases are not actively recruited to the site after transcription repression. Instead, H3K36 demethylation could occur as part of routine maintenance of chromatin, without targeted recruitment of demethylases to any particular site. This model fits with the previous results showing that only low levels of Jhd1, Rph1, and Gis1 can be detected by ChIP assay on transcriptionally active or inactive loci [21]. Although the H3K36me3 was detectable in recently transcribed locus for about 60 minutes, it is relatively short period compared to turnover of H3K4me3 in promoter regions, which has been shown to last about five hours [29]. This suggests that H3K4me3 could be actively maintained for a couple of cell divisions to “memorize” active promoters, while the H3K36me3 mark is not actively preserved and is re-established during active transcription in a RNAPII-dependent manner. We found that deletion of H3K36-specific demethylases led to prolonged turnover of H3K36me3, indicating their role in the process (Fig. 2). Although the H3K36me3 signal decreased to basal levels in both wt and mutant strains after 120 minutes of transcription repression, the rate of signal disappearance in mutant strains was slightly lower. We estimate that the half-life of the H3K36me3 after transcriptional inhibition in wild-type cells is about 20–30 minutes compared to 30–60 minutes in demethylase deletion strains. Interestingly, we could not detect any significant increase of H3K36 methylation in actively transcribed gene loci in any of the demethylase deletion strains, indicating again that H3K36 demethylases are not directly targeted to transcribed loci to balance or restrict the action of Set2. Also previous studies have shown that deletions of H3K36 specific demethylases have minimal effect on the overall level of histone methylation [21, 23, 24]. Replacement of modified histones with unmodified versions would be an alternative way to erase chromatin marks. To elucidate the role of replication-dependent exchange of histones in erasure of H3K36 methylation, we followed H3K36me3 turnover in G1-arrested cells. In cell-cycle arrested cells the H3K36me3 mark was maintained for prolonged time, suggesting that in normal circumstances the replication-coupled exchange of histones accelerates the removal of H3K36me3 from chromatin. Importantly, H3K36me3 modification was remarkably stable in G1-arrested cells in the absence of H3K36 demethylases (Fig. 3). Based on these observations we favour the model that the H3K36me3 mark is removed from transcribed loci in collaboration of passive action of histone demethylases and replication-coupled exchange of histones.

## Supporting Information

S1 Fig Demethylase deletion strains exhibit similar phenotype as wild type strain. (EPS)

S2 Fig Cell cycle profiles of G1-arrested wt and *rph1Δjhd1Δgis1Δ* strains. (EPS)

S1 Table List of qPCR primers. (DOCX)
