# Supplementary material for: Distribution and Maintenance of Histone H3 Lysine 36 Trimethylation in Transcribed Locus
Source: PLoS One. 2015 Mar 16;10(3):e0120200. doi: 10.1371/journal.pone.0120200 (PMC4361658; doi:10.1371/journal.pone.0120200)
Supplement: S1 Table — (DOCX) [file pone.0120200.s003.docx]

| **Primers** | **Sequence** |
| --- | --- |
| *VPS13* 2.6 kb  Forward  Reverse | ACGTTAATTACTCTTCTGGTTCCGA  AGAATGCTATTTTCGCCTGATCGAT |
| *VPS13* 3.0 kb  Forward  Reverse | TGATTCTATAAAGCTGGCAACGT  CTAAATACCGAATCCCTGGAAAA |
| *VPS13* 3.6 kb  Forward  Reverse | GTACAACCAAGCCCCTTCCAT  ACTCACCCAAATAAAACCGCAT |
| *VPS13* 4.0 kb  Forward  Reverse | TGAATCAGGAGGAAAAGGCGA  AAGTGACGCCCTTTGTCTTGTTA |
| *Tel6-R*  Forward  Reverse | TAACAAGCGGCTGGACTACTTTC  GATAACTCTGAACTGTGCATCCACTC |

**S1 Table.** *List of qPCR primers.*
